# Obesity-Associated Differentially Methylated Regions in Colon Cancer

**DOI:** 10.3390/jpm12050660

**Published:** 2022-04-20

**Authors:** John J. Milner, Zhao-Feng Chen, James Grayson, Shyang-Yun Pamela Koong Shiao

**Affiliations:** 1 College of Nursing, Augusta University, Augusta, GA 30912, USA; 2 Bachelor Degree Program in Pet Healthcare, Yuanpei University of Medical Technology, Hsinchu 30015, Taiwan, China; ivan.chen1966@gmail.com; 3 Hull College of Business, Augusta University, Augusta, GA 30912, USA; jgrayson@augusta.edu; 4 Medical College of Georgia, Augusta University, Augusta, GA 30912, USA; pshiao@msn.com

**Keywords:** differentially methylated regions, colon cancer, obesity, biomarkers, generalized regression

## Abstract

Obesity with adiposity is a common disorder in modern days, influenced by environmental factors such as eating and lifestyle habits and affecting the epigenetics of adipose-based gene regulations and metabolic pathways in colorectal cancer (CRC). We compared epigenetic changes of differentially methylated regions (DMR) of genes in colon tissues of 225 colon cancer cases (154 non-obese and 71 obese) and 15 healthy non-obese controls by accessing The Cancer Genome Atlas (TCGA) data. We applied machine-learning-based analytics including generalized regression (GR) as a confirmatory validation model to identify the factors that could contribute to DMRs impacting colon cancer to enhance prediction accuracy. We found that age was a significant predictor in obese cancer patients, both alone (*p* = 0.003) and interacting with hypomethylated DMRs of *ZBTB46*, a tumor suppressor gene (*p* = 0.008). DMRs of three additional genes: *HIST1H3I* (*p* = 0.001), an oncogene with a hypomethylated DMR in the promoter region; *SRGAP2C* (*p* = 0.006), a tumor suppressor gene with a hypermethylated DMR in the promoter region; and *NFATC4* (*p* = 0.006), an adipocyte differentiating oncogene with a hypermethylated DMR in an intron region, are also significant predictors of cancer in obese patients, independent of age. The genes affected by these DMR could be potential novel biomarkers of colon cancer in obese patients for cancer prevention and progression.

## 1. Introduction

Obesity, or excess adiposity and having a body mass index (BMI) ≥ 30, is a preventable and treatable condition, with lifestyle and environmental modifications, which has tripled since 1975 and now affects approximately 13% of the world population [1]. Obesity has been linked to tumorigenesis and cancer progression in various organs and a reduction in life expectancy by up to 14 years [2,3,4,5,6,7]. Mortality is increased up to 10% when obesity is present with colorectal cancer (CRC), whereas the risk of CRC is reduced up to 21% with a decrease in BMI and changes in lifestyle [8,9].

The risk of developing CRC increases with age, and 50–80% of CRC can be attributed to epigenetic changes due to lifestyle and BMI [10,11,12,13,14], with increased BMI as a risk factor in the proliferation of CRC [15,16,17,18,19]. CRC is the second most common cause of cancer death in the United States, with approximately 53,000 deaths occurring in 2021 [20]. For personalized medicine, it is clinically imperative to understand the impact of obesity on epigenetic changes to prevent the progression of CRC.

DNA methylation (DNAm) is an epigenetic regulation of gene function that is implicated in the formation of CRC [21,22,23,24] and is impacted by obesity [25,26,27,28] and age [29,30,31,32]. DNAm occurs when a methyl group attaches to a cytosine nucleotide, usually within a CpG dinucleotide (CpG), inhibiting the expression of the nucleotide, thereby potentially altering the expression of the gene itself [33]. Differentially methylated regions (DMRs) represent groups of methylated cytosines within close range in various tissue types and developmental stages [34]. DMRs located in the promoter region (first exon of a gene) were linked to gene silencing [35,36,37,38,39], and the position of DMRs related to the transcription start site (TSS) could impact transcription and gene function [40].

Hypomethylation (a reduction in methylated CpG) was associated with increased expression of gene function, whereas hypermethylation with decreased expression may potentially lead to chronic diseases including cancer, especially if methylation occurs in the promoter region of a gene [37,41,42,43,44,45]. Hypermethylated tumor suppressor genes (TSG) could lead to dysfunctional gene division or apoptosis, leading to abnormal cell growth; whereas hypomethylation with oncogenes causes cells to divide abnormally faster [46]. Hypermethylation and reduction in TSG function of *SRGAP2C* (Slit-Robo Rho GTPase-activating protein 2C) [47,48,49] and *ZBTB46* (zinc finger and born-to-bind domain containing 46) [50,51,52] were associated with tumorigenesis and cancer metastasis. Conversely, hypomethylation and upregulated oncogene functions of HIST1H3I (Histone linker 1 with Histone H3.1), HIST1H3D (Histone linker 1 with Histone H3.D) [53,54,55,56], NFATC4 (Nuclear factor of activated T-cells cytoplasmic 4) [57,58,59,60] and HOXB8 (Homeobox B8) [61,62,63] were associated with adiposity and colon cancer.

Significant research is being conducted on methylation of CpG and DMR in colon cancer [64,65,66], yet there is little consensus on what constitutes a significant methylation threshold that could potentially translate to clinical significance. Whether the methylation threshold is purely statistical using *p* values, or if it is a differential change measured in a percent difference, has not yet been sufficiently documented or validated. Many studies focus on single-gene associations with methylated CpG or DMR, some taking clinical data into context [67,68]. The genomic region of DMR and the impact on gene expression has also been studied, showing that DMRs on promoter regions adversely affect gene function [38,39]. With advanced sequencing technology and machine-learning-based analytics [69,70,71], we conducted this study to examine DMRs in association with obesity as a significant contributing factor for colon cancer prevention.

The United States Centers for Disease Control and Prevention (CDC) has established a need for increasing precision in cancer prevention [72]. Precision medicine takes individual differences in lifestyle, environment, and biology into account, requiring complex interactive analysis and predictive analytics, as well as standardized coding [73,74,75]. Therefore, we accessed data from the Cancer Genome Atlas (TCGA) to evaluate the association of obesity in human colon tissue to locate DMR-associated genes of interest to examine the associations of obesity with colon cancer [76]. We then applied groundbreaking machine-learning-based predictive analysis to locate DMRs, integrating BMI and age into the validation models, to enhance the accuracy of prediction.

## 2. Materials and Methods

### 2.1. Demographic and Methylation Data

We obtained methylation data files from TCGA Colon Cancer project (COAD) version 1.23.0 (https://portal.gdc.cancer.gov, accessed 16 June 2018) that were filtered to include harmonized Illumina 450 K methylation data, BMI, age and gender from normal colon (*n* = 15) and colon cancer (*n* = 225) tissues using an R package designed for data retrieval, grouping and DMR analysis, TCGAbiolinksGUI [77,78,79,80]. The Illumina 450 K methylation array provided data on 485,000 CpG sites, which covered approximately 1.6% of all CpG sites, (0.01% of the entire genome), and methylation information on 99% of all known genes [81,82,83,84]. Data included three groups of non-obese control (no cancer), non-obese cancer (BMI < 30) and obese cancer (BMI ≥ 30). At the time of data retrieval, this comprised the entire list of cases that met the inclusion criteria. BMI was used as a grouping independent variable and age as an independent variable in the regression model.

### 2.2. DMR Bioinformatics Analysis

CpG site analysis and DMR identification were completed using additional R packages limma (v. 3.34.9, February 2018) and bumphunter (v. 1.20.0, November 2012). Limma involves a matrix-type schema to analyze intra-sample variability (*n* = 240) per individual CpG site (*n* ≈ 485,000), and Bayesian corrected *p* values were provided between groups [85]. Bumphunter includes linear regression and permutation testing to determine clusters of DMR with significant CpG sites [86,87]. DMR with >2 CpG sites, excluding sex chromosomes and having ≥5% proportional change between groups of obese and non-obese cancer were annotated to protein-coding genes. A 3–10% difference in DNAm level between groups was noted as significant [88,89,90,91].

Gene annotation was conducted using data from the Catalogue of Somatic Mutations in Cancer (COSMIC) v86 August 2018 (https://cancer.sanger.ac.uk/cosmic (accessed on 16 June 2018)) [92,93] and the University of California, Santa Cruz (UCSC) genome browser (GRCh38/hg38, December 2013) (www.genome.ucsc.edu (accessed on 16 June 2018)) [94]. Gene ontology and pathway analysis were conducted using the Database for Annotation, Visualization, and Integrated Discovery (DAVID) v 6.8 (https://david.ncifcrf.gov (accessed on 16 June 2018)) [95]. To isolate genes related to obesity, only genes in both obese and non-obese comparison groups, and only those that could be further annotated to cancer and/or adipose-related functions through ontology or pathway analysis, were considered.

### 2.3. Predictive Analysis

Generalized regression (GR) was performed using JMP Pro v. 14 (SAS Institute, Cary, NC, USA). as a machine-learning tool to determine a predictive model from the genes identified in the obese and non-obese cancer groups. To create the predictive model for GR, variables were recoded into dichotomous values based on median distribution across the variable. The model created a prediction profile for associations between the parameters of interest and the strength of the parameters within the predictive model. Unlike logistic regression (LR), which assumed that all variables share a linear association, GR performed analysis on each independent variable to determine associations with the dependent variable and created a model that applied the nonlinear association to each variable in the prediction [96,97,98], which was then compared to the (LR) model for validation [99,100].

The final GR model was derived based on several criteria, including Akaike information criterion (AICc), misclassification rate and the area under the receiver operating characteristic (ROC) curve (AUC). For internal validity of the predictive model, an AUC as close to 1 (100%) is desired. Sensitivity refers to the number of actual cases with the finding of a positive result, whereas specificity is the number of actual cases without the finding of a negative result. AUC is a method to plot the sensitivity and specificity of test results to determine the accuracy of true positives versus false negatives [101]. Misclassification rate is precision of the model by calculating the number of errors by the total number of observations. Ideally, this number should be low. AICc is an estimate of the fitness of the model and should also be a low number.

To establish a GR model, 10 genes with the highest differential methylation, both hypomethylated and hypermethylated, were analyzed using interaction profilers and regression algorithms. The prediction validation was developed using a GR adaptive elastic net and Leave One Out (LOO) methodology with an 85% training/15% validation proportion created for machine-learning-based iterations. Interactions between factors were examined using interaction profiler plots between parameters of genes or with age, with steps of iterations to eliminate parameters from the model that had no significance or altered the significance of other parameters and prediction accuracy. Elastic net models presented a higher sensitivity and specificity than lasso [102,103,104], and LOO methodology was used to eliminate insignificant parameters in the model and is suitable for analysis with smaller sample sizes [105,106].

## 3. Results

Demographic factors among control and two cancer groups of obese and non-obese were compared (Table 1). BMI was different between obese cancer and two other groups (*p* < 0.0001), and cancer groups were younger in age (*p* < 0.05), with obese cancer being 9.7 years younger and non-obese cancer being 7.7 years younger than the control group on average. There were no differences among three groups on gender and racial distributions.

### 3.1. Significant DMRs and Associated Genes between Groups

DMR analysis was performed to determine the number of protein-coding genes of significance between groups, using a 5%, 10% and 15% methylation change between groups (Table 2), which shows a complete list of DMRs with the highest methylation differences for three between-groups pairs. DMR coordinates and gene functions are provided in the supplementary tables (Tables S1 (hypomethylated) and S2 (hypermethylated). To test the hypothetical association between obesity and CRC, gene ontology was performed with a list of 518 genes comprising a 5% methylation change in both hypermethylated (n = 178) and hypomethylated (n = 340) genes between the obese and non-obese cancer groups. A 5% methylation difference was used due to the need for a sufficient list of genes for ontological analysis between the obese and non-obese cancer groups. No novel pathways with statistical significance were discovered between the obese and non-obese cancer groups; therefore, further ontological analysis was not conducted.

Table 3 shows 10 DMRs with the highest hypomethylation difference between obese and non-obese cancer groups, and Table 4 shows the 10 DMRs with the highest hypermethylation between the two groups. Genes with functions linked to adiposity or glucose metabolism, cancer-related functions, and both adiposity and cancer-related functions were noted. Genes noted in Supplemental Tables S1 (hypomethylated) and S2 (hypermethylated) were used to derive the final GR model.

### 3.2. Significant Predictors

The most significant predictors associated with obesity between the two cancer groups were age ≥ 76 (*p* = 0.004); *HIST1H3I*, a hypomethylated oncogene (*p* = 0.002); *NFATC4*, a hypermethylated oncogene (*p* = 0.027); *SRGAP2C*, a hypermethylated tumor suppression gene (*p* = 0.025); and *ZBTB46*, a hypomethylated TSG interacting with age (*p* = 0.024), which was the only gene to have significant interaction with age in our prediction model. Variable importance analysis using independent uniform inputs shows that the order of variable importance is age (total effect (TE): 0.374), *HIST1H3I* (TE: 0.299), *ZBTB46* (TE: 0.295), *SRGAP2C* (TE: 0.184), *NFATC4* (TE: 0.156), *HOXB8* (TE: 0.098) and *HIST1H3D* (TE: 0.024). Genes and interactions left in the model without significance were left to protect the integrity of the model itself, as the removal of these predictors caused model instability (see Supplemental Figure S1).

**Figure 1:**
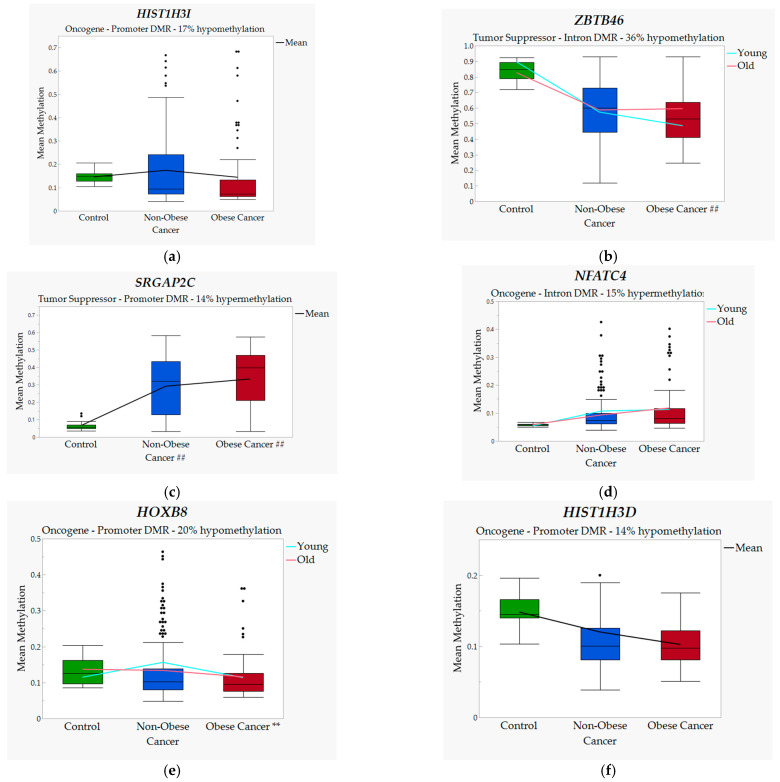
Methylated genes from the generalized regression model. Notes. Genes with age interaction show mean values by age as trendlines; genes without interaction with age show overall mean trendline. Hypomethylation and hypermethylation percent indicates change between obese and non-obese groups. Genes are ordered (**a**–**f**) based on total effect from generalized regression model; ## indicates *p* < 0.05 for cancer group to control; ** indicates *p* < 0.05 between obese and non-obese cancer.

*HIST1H3I* is an oncogene located on chromosome 6 that encodes a nuclear protein responsible for nucleosome structure and histone modification. It has been shown to have a high affinity for tumorigenesis, has been identified as a potential biomarker for CRC, and has been isolated in adipocytes [53,54,107]. Our data (see Figure 1a) show the promoter region DMR of *HIST1H3I* to have 17% hypomethylation between obese and non-obese cancer, and GR analysis showed no interaction between *HIST1H3I* and age in the prediction model. Mean values appear to increase from control group due to outliers in the data samples, but GR is based on median values, which are not represented by outliers.

TSG such as *ZBTB46*, a zinc finger/BTB domain protein gene on chromosome 20, represses the oncogene *PRDM1* and has similar functions to autoimmune regulators [50,51,52]. Although located on an intron, the DMR affecting *ZBTB46* showed a 36% reduction in methylation between obese and non-obese cancer groups (see Figure 1b), and GR analysis showed significant interaction with age (*p* = 0.024).

*SRGAP2C* is a SLIT-ROBO GTPase-activating tumor-suppressing gene located on chromosome 1. Changes in expression may contribute to cancer metastasis, and the *SRGAP2* protein is reduced or absent in many tumor samples [47,48,49]. The promoter-region DMR on *SRGAP2C* showed significant hypermethylation between all three groups (see Figure 1c), with the lowest at 14% proportional change between the obese and non-obese cancer group, and it revealed a marginal interaction with age in the GR model.

*NFATC4*, an oncogene located on chromosome 14, encodes a protein from the nuclear factor of an activated T-cell family, which is a DNA-binding complex, is expressed in many cancer tissues, and has been shown to enhance tumorigenesis. With obesity, *NFATC4* is known to initiate inflammatory processes, and it is associated with increased cell death in older patients [57,58,59,60]. The significant DMR for *NFATC4*, located on an intron, had 15% hypermethylation between obese and non-obese cancer (see Figure 1d), and *NFATC4* had a marginal interaction with age in the GR model. A large number of outliers appears to minimize the mean value differential for *NFATC4*, but these outliers are not factored into the GR model.

*HOXB8* located on chromosome 17 is a known oncogene that is associated with colorectal cancer. It is downregulated in colon cancer, but downregulation has been associated with favorable prognosis in renal cancer [61,62,63,108]. *HOXB8* has promoter-region DMR with a 20% hypomethylation between groups (see Figure 1e), and a marginal interaction with age in the prediction model.

*HIST1H3D*, similar to *HIST1H3I*, is a known oncogene also located on chromosome 6, functioning as a chromatin compactor. It is upregulated in cancer, and a reduction in its expression causes chromatin structure closing [55,56,109]. Figure 1f shows the *HIST1H3D* promoter-region DMR having a 14% hypomethylation between obese and non-obese cancer, with no significant age interaction in the GR model.

**Figure 2:**
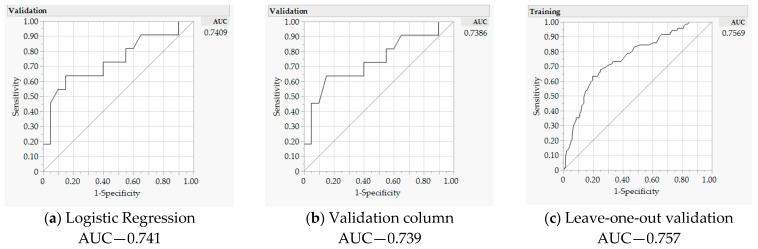
Receiver operating characteristic (ROC) curve and area under the curve (AUC) for logistic and generalized regression with adaptive elastic net. (**a**) represents logistic regression, (**b**) is adaptive elastic net with validation column, and (**c**) is adaptive elastic net with leave-one-out validation.

The prediction model shown in Table 5 presents comparable misclassification rates, with 29% for validation, 27% for LOO and 29% in the LR models, with equal precisions. AICc in the validation model was 71 compared to 76 in the LR model, revealing a fitter validation model. Figure 2 shows that the AUC for LR (a) was 74%, revealing similar internal validity to our GR model with 74% AUC for validation (b) and 76% for LOO (c). GR models provide higher quality predictions than LR models in locating possible obesity-associated colon cancer biomarkers.

## 4. Discussion

Generalized regression is a powerful machine learning tool to capture associations between variables, rather than assuming that one causes the other. GR eliminates variables that have no effect on the final model, allowing the remaining variables to have a greater effect on the overall model. Using GR, we reduced the pool of colon cancer obesity-impacted DMR-associated genes to six (6) and determined that age was an associated variable when considered independently, also when interacting with several genes, most notably *ZBTB46* (*p* = 0.024). We further determined that neither gender nor ethnicity were significant factors, and this is one of only a few studies to use GR in a methylation study that also used a differential methylation value of DMR.

Of the list of top 10 genes containing the DMR with the largest methylation difference, *HOXB8* was the only gene with a statistically significant hypomethylated DMR in the obese to non-obese group (*p* = 0.026). As an oncogene, increased expression is associated with colorectal cancer. Two genes had statistically significant DMR hypermethylation in the obese to non-obese group: *ZNF426* (*p* = 0.049), a zinc finger protein-coding gene involved in transcriptional regulation; and *TUBB3* (*p* = 0.043), a beta-tubulin protein family coding gene that plays a role in axon guidance. When used in the GR model, only HOXB8 had borderline significance, which caused model instability when it was removed.

One limitation of this study was the use of methylation data from solid tumor tissue, rendering it difficult to generalize for biomarker analysis; however, it provides significant information about the genomic mechanisms impacted by obesity that may be targeted by precision medicine for colon cancer patients. Further study needs to be conducted to compare methylation changes in both solid tissue and body fluid samples, to determine whether DNAm occurs systemically or is isolated to the cancer tissue, and further study needs to be conducted to determine the level that DNAm impacts the function of the gene, whether at an individual CpG site or a DMR, such that future studies can all start from a leveled analysis point. The use of a single cohort cancer database is another limitation of this study, which will be reduced in a follow-up study by using multiple cohorts as well as survival analysis to validate these findings.

Using machine-learning-based tools and grouping colon cancer cases based on BMI, we have identified genes associated with obesity, related to lifestyle that may modified, potentially impacting colon cancer through methylation. *HIST1H3I*, *ZBTB46*, *SRGAP2C* and *HIST1H3D* are all potential novel biomarkers identified through our analysis method, and using GR, multiple genes impacted by DMR can be identified with cofactors from patient lifestyle. DNAm analysis and interpretation are becoming easier and less expensive to perform and can provide insights into disease processes never considered feasible during treatment processes in the past. Combining DNAm with GR provides precision-medicine-based healthcare the tools necessary to focus on patient-centered treatment for cancer and chronic diseases.

## Figures and Tables

**Table 1:** Demographic characteristics of the sample cases.

		Cancer	
Mean ± SD (Range)	Control (*n* = 15)	Non-Obese (*n* = 154)	Obese (*n* = 71)
BMI, Kg/m^2^	25.5 ± 2.7 (20.1–29.8)	24.9 ± 3.1 * (14.7–29.8)	36.1 ± 2.8 * (30.0–54.1)
Age, years	81.7 ± 13.7 (48–102)	74.0 ± 15.1 * (41–106)	72.0 ± 12.0 * (38–92)
Gender Female (%)	9 (60)	67 (43.5)	37 (52)
Race White (%)	13 (87)	116 (75)	49 (69)
Black (%)	2 (13)	30 (19)	21 (30)
Other (%)	0	8 (6)	1 (1)

Notes. BMI: Body Mass Index; * indicates *p* < 0.05.

**Table 2:** Summary of differentially methylated regions (DMR) with unique protein-coding genes per group comparison.

Groups	Non-Obese Cancer/Control		Obese Cancer/Control		Obese Cancer/Non-Obese Cancer	
Differential Methylation	Hyper	Hypo	Hyper	Hypo	Hyper	Hypo
5%	4270	3744	4203	4073	178	340
10%	2967	1644	2876	1909	25	48
15%	2248	637	2173	828	6	10

Notes. Hypo refers to hypomethylated; Hyper refers to hypermethylated.

**Table 3:** Genes associated with top hypomethylated differentially methylated regions (DMR) between obese and non-obese cancer groups.

	DMR		Dis to TSS	DNAm		Gene Function
Gene	# CpG	Region		Non-Obese	Obese	
*HIST3H2A* ^‡^	25	Promoter	860	8.61	6.80	DNA repair, MMR
*HIST3H2BB* ^‡^	25	Promoter	701	8.61	6.80	DNA repair, MMR
*HOXB8* ^†^	18	Promoter	−279	14.47	11.48	Oncogene
*HIST1H3I* ^‡^	11	Promoter	−24	17.43	14.47	Oncogene
*TUBB2A* ^‡^	3	Intron	−593	9.93	8.26	GTP binding
*TMCO1* ^‡^	13	Promoter	210	5.49	4.59	Calcium homeostasis
*PRAC2* ^†^	4	Promoter	109	10.32	8.63	Oncogene
*AMOTL2* ^‡^	4	Intron	−10,235	15.79	13.38	Inhibits Wnt pathway
*ARL4D* ^^^	13	Promoter	107	8.03	6.82	Suppresses adipogenesis
*HIST1H3D* ^‡^	13	Promoter	59	12.02	10.26	Oncogene

Notes: # CpG—number of methylated CpG sites; Dis to TSS—distance (in base pairs) to transcription start site from DMR start; DNAm—mean methylation percent; ^^^ represents genes that can be annotated to adiposity or glucose-related functions, ^†^ to cancer-related functions, and ^‡^ to both cancer and adipose/glucose-related functions.

**Table 4:** Genes associated with top hypermethylated differentially methylated regions (DMR) between obese and non-obese cancer groups.

	DMR		Dis to TSS	DNAm		Gene Function
Gene	# CpG	Region		Non-Obese	Obese	
*GNPDA2* ^^^	12	Promoter	107	7.86	9.74	Protein metabolism
*LSM14A* ^†^	9	Promoter	540	3.44	4.12	Immune response
*ZNF426* ^†^	11	Promoter	107	21.70	25.80	Transcription regulation
*NFATC4* ^‡^	8	Intron	−466	9.98	11.52	Oncogene
*ZNF852* ^†^	3	Promoter	31	2.81	3.24	Transcription regulation
*FAM72B* ^†^	9	CDS	3021	29.19	33.34	Oncogene
*SRGAP2C* ^‡^	9	Promoter	879	29.19	33.34	Tumor Suppression Gene
*TNFAIP2* ^†^	3	Promoter	445	14.28	16.10	Mediator of inflammation
*ZNF747* ^†^	7	Promoter	188	7.18	8.07	Transcription regulation
*TUBB3* ^‡^	3	Intron	2448	6.36	7.14	Oncogene, immune response

Notes: # CpG—number of methylated CpG sites; CDS—coding DNA sequence; Dis to TSS—distance (in base pairs) to transcription start site from DMR start; DNAm—mean methylation percent; ^^^ represents genes that can be annotated to adiposity or glucose-related functions, ^†^ to cancer-related functions, and ^‡^ to both cancer and adipose/glucose-related functions.

**Table 5:** Predictors of obesity-associated differentially methylated regions in colon cancer.

			Generalized Regression Adaptive Elastic Net			
	Logistic Regression		Leave-One-Out		Validation Column	
Parameters	Estimate	*p* (χ^2^)	Estimate	*p* (χ^2^)	Estimate	*p* (χ^2^)
Intercept	−0.1997	0.738	0.0114	0.984	−0.2869	0.613
Age (≥76)	−2.4211	0.004	−2.3076	0.003	−2.2997	0.004
*HIST1H3I* (hypo, promoter) ^‡^	1.1541	0.003	1.2026	0.001	1.1243	0.002
*NFATC4* (hyper, intron) ^‡^	−1.1034	0.046	−1.3256	0.006	−1.1133	0.027
*SRGAP2C* (hyper, promoter) ^‡^	−1.2821	0.026	−1.4779	0.006	−1.2355	0.025
Age * *ZBTB46*	1.7993	0.020	1.8545	0.008	1.7343	0.024
Age * *NFATC4*	1.3051	0.078	1.1752	0.078	1.3065	0.069
Age * *HOXB8*	1.0252	0.163	1.1410	0.084	0.9161	0.081
Age * *SRGAP2C*	1.0565	0.153	1.1584	0.088	1.0062	0.168
*HIST1H3D* (hypo, promoter) ^‡^	−0.3225	0.401	−0.6169	0.089	−0.3119	0.419
*ZBTB46* (hypo, intron) ^†^	−0.2551	0.637	−0.2889	0.550	−0.2036	0.702
*HOXB8* (hypo, promoter) ^†^	−0.0984	0.856	0.0115	0.979	0.0000	1.000
Misclassification rate	0.290	-	0.277	-	0.290	-
AICc	76.63	-	-	-	71.067	-
Area under the curve	0.741	-	0.757	-	0.739	-

Notes—data not available; AICc—Akaike’s information criterion with corrections; * denotes interaction. ^†^ represents genes that can be annotated to cancer-related functions and ^‡^ to both cancer and adipose/glucose-related functions.

## Supplementary Materials

The following supporting information can be downloaded at: https://www.mdpi.com/article/10.3390/jpm12050660/s1, Figure S1: Interaction profile matrix for generalized regression model parameters, Table S1: Genes associated with top hypomethylated differentially methylated regions (DMR) between groups, Table S2: Genes associated with top hypermethylated differentially methylated regions (DMR) between groups. Original TCGA methylation files: demographics.csv, annotations.csv, methylation_betas.csv.
