# Supplementary material for: Obesity-Associated Differentially Methylated Regions in Colon Cancer
Source: J Pers Med. 2022 Apr 20;12(5):660. doi: 10.3390/jpm12050660 (PMC9142939; doi:10.3390/jpm12050660)
Supplement: Supplementary file 1 [file jpm-12-00660-s001.zip › jpm-1645163-supplementary.pdf]

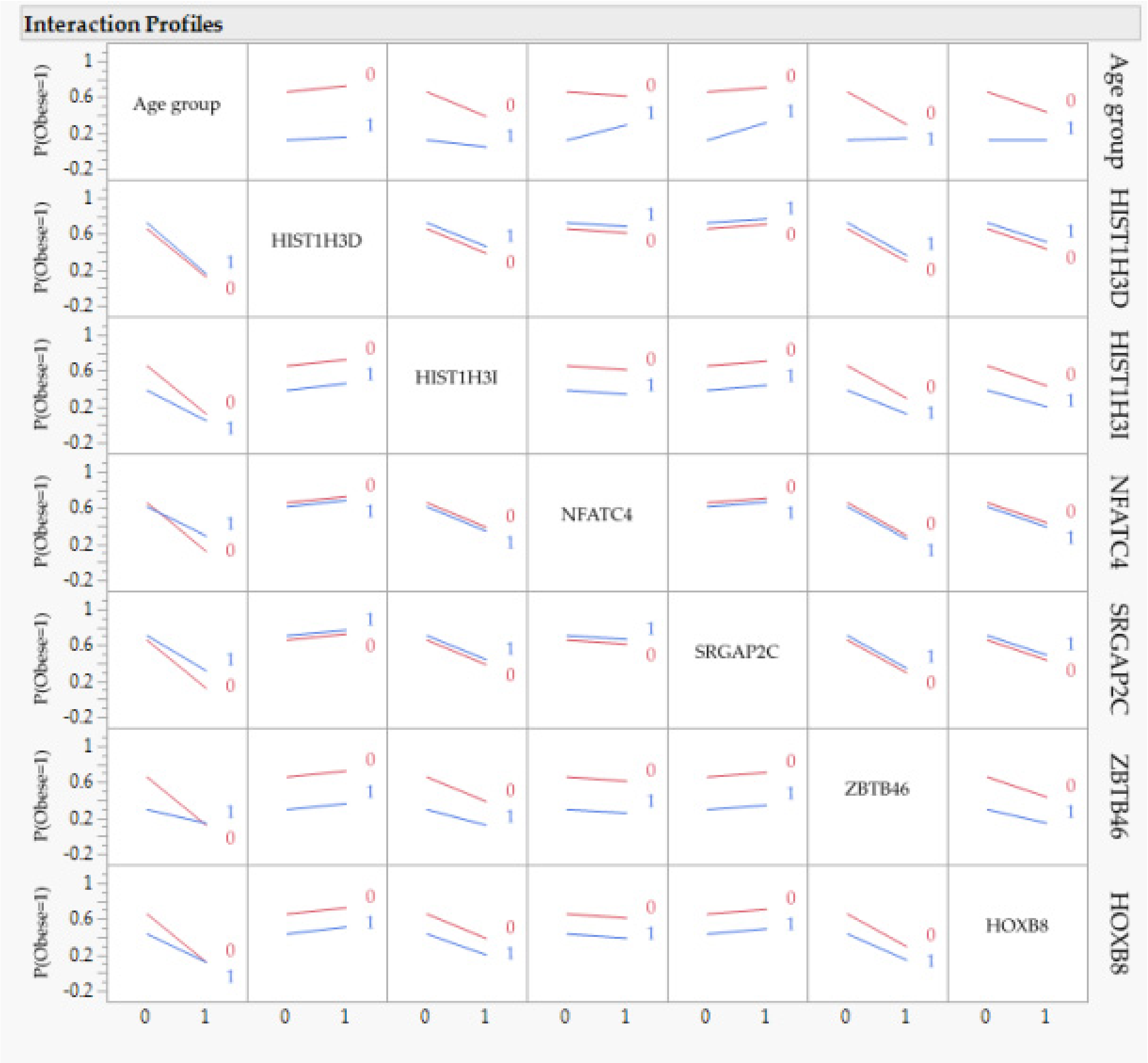

Figure S1. Interaction profile matrix for generalized regression model parameters. Note: age group 0 = below median (younger). Red lines indicate younger cases; blue lines indicate older.

Table S1. Genes associated with top hypo-methylated differentially methylated regions (DMR) between groups.

Group	Gene	# CpG	DMR Location			Dis. to	Methylation				Gene Function
			Coordinate	Region	Str	TSS	CTL *	NOB *	OB *	*p*	
NOB/ CTRL	*RUBCN*[f]	12	chr3:197736464-197737273	Promoter	-	554	13.64	5.96	-	<0.001	Impairs the autophagosome maturation process
	USP4[g]	11	chr3:49339907-49340421	Promoter	-	350	20.48	10.67	-	<0.001	Protein metabolism, interacts with ADORA2A
	ZNF852[◊]	3	chr3:44510396-44510667	Promoter	-	31	5.18	2.81	-	0.002	Transcription regulation, may be a pseudogene
	RTEL1[e]	4	chr20:63687006-63687305	Intron	+	−29,196	38.81	21.38	-	<0.001	Known to protect telomeres during DNA replication
	CIITA[a]	9	chr16:10878925-10879448	Intron	+	−1727	37.81	21.57	-	<0.001	Positive regulator of class II major histocompatibility complex
	HIST1H3J	5	chr6:27890388-27890786	Promoter	-	−6	17.85	10.65	-	0.013	Transcription regulation, DNA repair, linked to alcoholism
	MST1R[d]	6	chr3:49902666-49902998	Promoter	-	−639	73.49	47.28	-	<0.001	Regulates cell survival, migration, and differentiation
	MRFAP1L1[c]	20	chr4:6709221-6710066	Promoter	-	186	23.03	15.00	-	<0.001	Interacts with MORF4L1 oncogene
	B3GNTL1	12	chr17:83051075-83052123	Promoter	-	313	31.42	20.53	-	<0.001	Protein metabolism, expressed in breast cancer
	GNG7[b]	6	chr19:2607727-2607969	Intron	-	−94,740	56.97	37.39	-	<0.001	Lipid anchor, GTPase activity, may inhibit insulin secretion
OB/ CTRL	*RUBCN*[f]	12	chr3:197736464-197737273	Promoter	-	554	13.64	-	5.60	<0.001	Impairs the autophagosome maturation process
	RTEL1[e]	4	chr20:63687006-63687305	Intron	+	−29,196	38.81	-	19.09	<0.001	Anti-recombinase functioning MMR
	USP4[g]	11	chr3:49339907-49340421	Promoter	-	350	20.48	-	10.71	<0.001	Protein metabolism, over-expressed in some cancer tissues
	CIITA[a]	9	chr16:10878925-10879448	Intron	+	-1727	37.81	-	21.61	<0.001	Tumor suppression (TSG)
	ZNF852[◊]	3	chr3:44510396-44510667	Promoter	-	31	5.18	-	3.24	0.012	Transcription regulation
	MST1R[d]	6	chr3:49902666-49902998	Promoter	-	−639	73.49	-	46.15	<0.001	Regulates cell survival, migration, and differentiation
	GNG7[b]	6	chr19:2607727-2607969	Intron	-	−94,740	56.97	-	35.90	<0.001	Lipid anchor, GTPase activity, may inhibit insulin secretion
	ZBTB46	3	chr20:63802584-63802850	Intron	-	−28,364	84.10	-	53.10	<0.001	Tumor Suppressor, transcription repressor
	NUDT7	9	chr16:77722519-77722784	Promoter	+	−5	12.02	-	7.65	<0.001	Lipid and fatty acid metabolism
	MRFAP1L1[c]	20	chr4:6709221-6710066	Promoter	-	186	23.03	-	14.70	<0.001	Interacts with MORF4L1 oncogene
OB/ NOB	*HIST3H2A*	25	chr1:228457406-228458733	Promoter	-	860	-	8.61	6.80	0.086	DNA repair, MMR
	HIST3H2BB	25	chr1:228457406-228458733	Promoter	+	701	-	8.61	6.80	0.086	DNA repair, MMR
	HOXB8	18	chr17:48613028-48614660	Promoter	-	−279	-	14.47	11.48	0.026	Oncogene
	HIST1H3I	11	chr6:27871920-27872291	Promoter	-	−24	-	17.43	14.47	0.174	Oncogene—linked to alcoholism
	TUBB2A	3	chr6:3156725-3156933	Intron	-	−593	-	9.93	8.26	0.127	Tubulin, beta 2A class IIa, GTP binding
	TMCO1	13	chr1:165768595-165769112	Promoter	-	210	-	5.49	4.59	0.413	Plays a key role in calcium homeostasis
	PRAC2	4	chr17:48723583-48723926	Promoter	+	109	-	10.32	8.63	0.304	Unknown function - expressed in colon and prostate CA
	AMOTL2	4	chr3:134364018-134364359	Intron	-	−10,235	-	15.79	13.38	0.313	Inhibits the Wnt/beta-catenin signaling pathway
	ARL4D	13	chr17:43398852-43399347	Promoter	+	107	-	8.03	6.82	0.236	Overexpression suppresses adipogenesis
	HIST1H3D	13	chr6:26196842-26197309	Promoter	-	59	-	12.02	10.26	0.063	Oncogene—linked to glioma and alcoholism

Note: Str—DNA strand; Dis to TSS—distance (in base pairs) to transcription start site from DMR start; *—mean methylation percent. Items in blue represent genes that can be annotated to adiposity or glucose-related ontologies, green to cancer-related ontologies, and red to both cancer and adipose/glucose-related ontologies. DMRs with matching superscript letters have significant methylation in more than one comparison group; ◊—gene has DMR with significant hyper- and hypo-methylation.

Table S2. Genes associated with top hyper-methylated differentially methylated regions (DMR) between groups.

Group	Gene	# CpG	DMR Location: Coordinate	DMR Location: Region	DMR Location: Str	Dis. to TSS	Methylation: CTL *	Methylation: NOB *	Methylation: OB *	*p*	Gene function
NOB/ CTRL	*ZNF625*[j]	8	chr19:12156183-12156871	Promoter	-	142	4.70	49.50	-	<0.001	Transcription regulation
	SLC6A15[f]	4	chr12:84911884-84912097	Intron	-	−779	6.33	44.91	-	<0.001	Transports neutral amino acids
	ZNF43[i]	5	chr19:21851592-21852141	CDS	-	15,933	6.57	40.47	-	<0.001	Transcription regulation
	RSPO3[e]	10	chr6:127118862-127119927	Promoter	+	−258	7.42	42.06	-	<0.001	Oncogene, regulation of Wnt pathway
	TMEFF1[h]	3	chr9:100473078-100474345	Promoter	+	35	3.31	17.87	-	0.011	May be a tumor suppressor
	GPM6A[d]	5	chr4:176001924-176002479	Promoter	-	147	8.09	41.80	-	<0.001	Regulation of endocytosis
	GALNT13[c]	3	chr2:153872431-153872815	Intron	+	−518	9.17	47.15	-	<0.001	Carbohydrate, protein, and metal ion metabolism
	ADHFE1[a]	10	chr8:66432262-66432754	Promoter	+	224	12.79	64.20	-	<0.001	Involved with TCA and cellular respiratory cycle
	FAM72B[b]	9	chr1:121183931-121187361	CDS	-	3021	6.62	29.19	-	<0.001	Unknown function- interacts with OTX1 gene in prostate cancer
	SRGAP2C[g]	9	chr1:121183931-121187361	Promoter	+	879	6.62	29.19	-	<0.001	TSG—cell migration; expressed in pancreas
OB/ CTRL	*ZNF625*[j]	8	chr19:12156183-12156871	Promoter	-	142	4.70	-	50.32	<0.001	Transcription regulation
	SLC6A15[f]	4	chr12:84911884-84912097	Intron	-	−779	6.33	-	42.98	<0.001	Transports neutral amino acids
	ZNF43[i]	5	chr19:21851592-21852141	CDS	-	15,933	6.57	-	40.67	<0.001	Transcription regulation
	RSPO3[e]	10	chr6:127118862-127119927	Promoter	+	−258	7.42	-	41.99	<0.001	Oncogene, regulation of Wnt pathway
	TMEFF1[h]	3	chr9:100473078-100474345	Promoter	+	35	3.31	-	18.13	0.013	May be a tumor suppressor
	GPM6A[d]	5	chr4:176001924-176002479	Promoter	-	147	8.09	-	42.41	<0.001	Regulation of endocytosis
	GALNT13[c]	3	chr2:153872431-153872815	Intron	+	−518	9.17	-	47.94	<0.001	Carbohydrate, protein, and metal ion metabolism
	ADHFE1[a]	10	chr8:66432262-66432754	Promoter	+	224	12.79	-	65.72	<0.001	Involved with TCA and cellular respiratory cycle
	FAM72B[b]	9	chr1:121183931-121187361	CDS	-	3021	6.62	-	33.34	<0.001	Unknown function- interacts with OTX1 gene in prostate cancer
	SRGAP2C[g]	9	chr1:121183931-121187361	Promoter	+	879	6.62	-	33.34	<0.001	TSG—cell migration; expressed in pancreas
OB/ NOB	*GNPDA2*	12	chr4:44726016-44726702	Promoter	-	107	-	7.86	9.74	0.202	Protein metabolism
	LSM14A	9	chr19:34172026-34173155	Promoter	+	540	-	3.44	4.12	0.088	Essential for formation of P-bodies, immune response
	ZNF426	11	chr19:9538258-9538734	Promoter	-	107	-	21.70	25.80	0.049	Transcription regulation
	NFATC4	8	chr14:24368666-24369118	Intron	+	−466	-	9.98	11.52	0.17	Oncogene—adipocyte differentiation
	ZNF852[◊]	3	chr3:44510396-44510667	Promoter	-	31	-	2.81	3.24	0.31	Transcription regulation
	FAM72B[b]	9	chr1:121183931-121187361	CDS	-	3021	-	29.19	33.34	0.08	Unknown function- interacts with OTX1 gene in prostate cancer
	SRGAP2C[g]	9	chr1:121183931-121187361	Promoter	+	879	-	29.19	33.34	0.08	TSG—cell migration; expressed in pancreas
	TNFAIP2	3	chr14:103123016-103123909	Promoter	+	445	-	14.28	16.10	0.441	Mediator of inflammation and angiogenesis
	ZNF747	7	chr16:30534203-30535192	Promoter	-	188	-	7.18	8.07	0.177	Transcription regulation
	TUBB3	3	chr16:89925727-89926552	Intron	+	2448	-	6.36	7.14	0.043	Oncogene, immune response, associated with Hedgehog

Note: Str—DNA strand; Dis to TSS—distance (in base pairs) to transcription start site from DMR start; *—mean methylation percent. Items in blue represent genes that can be annotated to adiposity or glucose-related ontologies, green to cancer-related ontologies, and red to both cancer and adipose/glucose-related ontologies. DMRs with matching superscript letters have significant methylation in more than one comparison group; ◊—gene has DMR with significant hyper- and hypo-methylation
